# 

**Published:** 1875-02-15

**Authors:** 


					﻿No. IV.
FEBRUARY IS, 1876.
Vol. XVI
THE
MEDICAL EXAMINER:
A.
Smi-lllonthto louvnal of ffledical mimes.
EDITED BY
N. S. DAVIS, M. D., and F. H. DAVIS, M. D.
Subscription. Price, Three Dollars per Annum, in advance.
Single Numbers, Fifteen Cents.
CHICAGO
PUBLISHED AT' 65 E. HANDOLPH ST'JREET'.
				

